# Comparative cytogenetics among *Boana* species (Anura, Hylidae): focus on evolutionary variability of repetitive DNA

**DOI:** 10.1590/1678-4685-GMB-2022-0203

**Published:** 2023-01-06

**Authors:** Sebastião Venancio, Rafael Bueno Noleto, Matheus Azambuja, Camilla Borges Gazolla, Bianca Rocha Santos, Viviane Nogaroto, Marcelo Ricardo Vicari

**Affiliations:** 1 Universidade Federal do Paraná, Centro Politécnico, Departamento de Genética, Programa de Pós-Graduação em Genética, Curitiba, PR, Brazil.; 2 Universidade Estadual do Paraná, Departamento de Biologia, União da Vitória, PR, Brazil.; 3 Universidade Estadual de Ponta Grossa, Departamento de Biologia Estrutural, Molecular e Genética, Ponta Grossa, PR, Brazil.

**Keywords:** Karyotype evolution, microsatellite, Neotropical treefrogs, rDNA

## Abstract

*Boana* comprises a diverse genus of Neotropical treefrogs, currently rearranged into seven taxonomic species groups. Although cytogenetic studies have demonstrated diversity in its representatives, the chromosomal mapping of repetitive DNA sequences is still scarce. In this study, *Boana albopunctata*, *Boana faber*, and *Boana prasina* were subjected to *in situ* localization of different repetitive DNA units to evaluate trends of chromosomal evolution in this genus. *Boana faber* and *B. prasina* had 2n=24 chromosomes, while *B. albopunctata* has 2n=22 and an intra-individual variation related to the presence/absence of one B chromosome. The location of 45S rDNA sites was different in the analyzed karyotypes, corroborating with what was found in the distinct phylogenetic groups of *Boana*. We presented the first description of 5S rDNA in a *Boana* species, which showed markings resulting from transposition/translocation mechanisms. *In situ* localization of microsatellite loci proved to be a helpful marker for karyotype comparison in *Boana*, commonly with cis accumulation in the heterochromatin. On the other hand, genomic dispersion of microsatellites may be associated with hitchhiking effects during the spreading of transposable elements. The obtained results corroborated the independent diversification of these lineages of species from three distinct phylogenetic groups of *Boana*.

## Introduction

Hylidae is a monophyletic group of treefrogs with 1,033 recognized species, which have undergone a progressive phylogenetic reorganization and are currently grouped into three subfamilies: Hylinae (747 sp.), Pelodryadinae (222 sp.), and Phyllomedusinae (67 sp.) (Frost, 2022). In addition, changes in the genera have been constant, *e.g.*, some species of the genus *Hyla* were relocated to the genus *Boana* (senior synonym of *Hypsiboas*) (Faivovich et al., 2005; Dubois, 2017). *Boana* (Hylinae) currently includes 99 species (Frost, 2022) rearranged into seven taxonomic species groups: *B. albopunctata*, *B. benitezi*, *B. faber*, *B. pellucens*, *B. pulchella*, *B. punctata*, and *B. semilineata* (Faivovich *et al.*, 2005, 2021; Wiens et al., 2005, 2010; Pyron and Wiens, 2011; Pyron, 2014). Based on shared morphological and molecular characteristics, these groups differ in the number of species and the arrangement of internal clades. *Boana albopunctata* and *B. faber* are members of the *B. albopunctata* and *B. faber* groups, respectively, while *B. prasina* is a member of the *B. pulchella* group with the largest number of species (Faivovich *et al.*, 2005, 2021).

Considering the cytogenetic descriptions available for *Boana*, the diploid number (2n) varies from 22 to 24, with karyotypes presenting a small variation in the fundamental number (FN) (Table 1). Most species of Phyllomedusinae and Pelodryadinae, recovered as the sister taxa of Hylinae, and share 2n=26 chromosomes, while a 2n=24 is considered a putative synapomorphy for Hylinae (Duellman, 2001; Faivovich et al., 2005, 2021; Ferro et al., 2018).

Despite the frequent 2n=24 chromosomes found in *Boana* spp., the karyotypic organization of the species cannot be considered conserved (Table 1). Most species share the nucleolus organizer regions (NORs) on small-sized chromosomes. However, the variation in this character has provided valuable phylogenetic evidence in some groups, like *B. albopunctata*, *B. pulchella*, and *B. semilineata* (Ferro et al., 2018). In addition, an intra- and inter-individual variation of the 0-1 B chromosome is observed in some *B. albopunctata* and *B. leucocheila* populations (Table 1).

**Table 1 -:** Cytogenetic data of species belonging to six different taxonomic groups of *Boana*.

Group	Species	Locality	2N	Chromosome pairs morphology												B	NORs	rDNAs location	References
				**1**	**2**	**3**	**4**	**5**	**6**	**7**	**8**	**9**	**10**	**11**	**12**				
*B. albopunctata*	*B. albopunctata*	São Paulo - Brazil	22	m	sm	sm	sm	sm	sm	m	sm	m	m	sm	--	--	--	--	Beçak (1968)
		Brazil	22	NI													--	--	Bogart (1973)
		São Paulo - Brazil	22+1	m	sm	sm	sm	sm	sm	sm	m	sm	m	m	--	m	8	--	Gruber et al. (2007)
		Goiás - Brazil	22	NI													--	--	Oliveira *et al.* (2012)
		Corrientes/Misiones - Argentina	22+(1-3)	m	m	sm	st	sm	sm	sm	m	m	sm	m	--	3m	8	18S - 8	Ferro et al. (2012)
		São Paulo - Brazil	22+1	NI													8	--	Gruber et al. (2014)
		Paraná - Brazil	22+1	m	m	sm	st	sm	st	sm	sm	sm	sm	m	--	m	8	5S - 2 18S - 8	Present study
	*B. cf. alfaroi*	Pará - Brazil	22	m	m	sm	sm	sm	sm	sm	m	m	sm	m	--	--	--	18S - 8	Ferro et al. (2018)
	*B. almendarizae*	Tungurahua - Ecuador	24	m	m	sm	sm	sm	sm	sm	m	sm	sm	sm	sm	--	12	--	Ferro et al. (2018)
	*B. calcarata*	Pastaza - Ecuador	24	m	m	sm	st	sm	sm	m	m	m	m	m	m	--	--	--	Ferro et al. (2018)
	*B. fasciata*	Huanuco - Peru	24	NI													--	--	Bogart and Bogart (1971)
	*B. heilprini*	--	24	m	m	sm	sm	m	sm	sm	m	sm	m	m	sm	--	11	--	Ferro et al. (2018)
	*B. lanciformis*	Amazonas - Brazil	22	m	sm	sm	st	st	st	m	m	sm	m	st	--	--	11	--	Mattos et al. (2014)
	*B. cf. lanciformis*	Amazonas - Brazil	24	m	m	sm	st	sm	st	sm	m	m	sm	sm	m	--	11	--	Ferro et al. (2018)
	*B. leucocheila*	Pará - Brazil	22+1	m	m	sm	sm	sm	sm	m	m	m	sm	m	--	m	8	18S - 8	Ferro et al. (2018)
	*B. multifasciata*	Goiás - Brazil	24	NI													--	--	Oliveira *et al.* (2012)
		Amazonas - Brazil	24	m	m	st	sm	st	st	sm	st	sm	m	m	m	--	11	--	Mattos et al. (2014)
		Pará - Brazil	22	m	m	sm	st	sm	st	sm	m	m	sm	m	--	--	8	18S - 8	Ferro et al. (2018)
	*B. raniceps*	--	24	m + sm													--	--	Rabello (1970)
		--	24	NI													--	--	Rabello et al. (1971)
		Mato Grosso do Sul - Brazil	24	m	sm	sm	sm	sm	sm	sm	m	sm	m	m	sm	--	11	--	Gruber et al. (2007)
		Goiás - Brazil	24	NI													--	--	Oliveira *et al.* (2012)
		Goiás - Brazil	24	NI													--	--	Gruber et al. (2014)
		Amazonas - Brazil	24	m	m	st	sm	sm	st	sm	m	m	st	m	st	--	11	--	Mattos et al. (2014)
		Misiones - Argentina	24	m	m	sm	st	sm	sm	m	m	sm	m	m	sm	--	11	18S - 11	Ferro et al. (2018)
*B. faber*	*B. albomarginata*	São Paulo - Brazil	24	m	sm	sm	sm	sm	a	sm	sm	m	m	m	m	--	--	--	Beçak (1968)
		Rio de Janeiro - Brazil	24	NI													--	--	Bogart (1973)
		Espírito Santo - Brazil	24	m	m	m	sm	sm	st	sm	sm	sm	m	m	m	--	2	--	Nunes and Fagundes (2008a)
		São Paulo - Brazil	24	m	m	m	sm	sm	sm	sm	m	m	m	m	m	--	3	18S - 2	Carvalho et al. (2009)
	*B. crepitans*	--	24	NI													--	--	Duellman and Cole (1965)
		--	24	m + sm (6 = a)													6	--	Rabello (1970)
		Argentina	24	NI													--	--	Bogart (1973)
		Alagoas - Brazil	24	m	sm	sm	sm	sm	sm	sm	m	sm	m	sm	sm	--	11	--	Gruber et al. (2007)
		Bahia - Brazil	24	m	m	sm	st	sm	sm	sm	m	m	sm	m	m	--	7	--	Carvalho et al. (2014)
	*B. faber*	Rio de Janeiro - Brazil	24	m	sm	sm	sm	sm	st	st	sm	m	m	sm	sm	--	--	--	Beçak (1968)
		Espírito Santo - Brazil	24	m	m	sm	st	sm	st	st	sm	m	sm	st	m	--	11	--	Nunes and Fagundes (2008a)
		São Paulo - Brazil	24	m	m	sm	sm	sm	sm	sm	m	m	m	m	m	--	11	18S - 11	Carvalho et al. (2009)
		São Paulo - Brazil	24	NI													11	--	Schmid and Steinlein (2016a)
		Misiones - Argentina	24	m	m	sm	st	sm	st	st	m	sm	sm	m	m	--	11	18S - 11	Ferro et al. (2018)
		Paraná - Brazil	24	m	m	sm	sm	sm	st	st	m	sm	m	sm	m	--	11	18S - 11 5S - 2	Present study
	*B. lundii*	Goiás - Brazil	24	NI													--	--	Oliveira *et al.* (2012)
	*B. pardalis*	Brazil	24	NI													--	--	Bogart (1973)
		Espírito Santo - Brazil	24	m	m	sm	st	sm	st	sm	sm	m	sm	m	m	--	11	18S - 11	Nunes and Fagundes (2008a)
	*B. rosenbergi*	--	24	NI													--	--	León (1970)
*B. pellucens*	*B. pellucens*	Esmeraldas - Ecuador	24	m	m	sm	sm	sm	sm	m	m	sm	m	sm	m	--	11	18S - 11	Ferro et al. (2018)
	*B. rufitela*	--	24	NI													--	--	Duellman (1967)
*B. pulchella*	*B. albonigra*	Jujuy - Argentina	24	m	m	sm	st	sm	st	sm	sm	m	m	m	m	--	11	--	Ferro et al. (2018)
	*B. bischoffi*	São Paulo - Brazil	24	m	sm	sm	sm	sm	sm	sm	sm	m	m	m	m	--	--	--	Beçak (1968)
		Sul do Brazil	24	m	m	sm	st	sm	st	sm	m	sm	sm	m	m	--	10	--	Raber et al. (2004)
		São Paulo - Brazil	24	m	m	sm	st	sm	st	sm	sm	m	m	m	m	--	11	--	Ferro et al. (2018)
	*B. caingua*	Misiones - Argentina	24	m	m	sm	st	sm	st	sm	m	m	m	m	m	--	12	--	Ferro et al. (2018)
	*B. callipleura*	Andes	24	NI													--	--	Duellman et al. (1997)
	*B. cipoensis*	Minas Gerais - Brazil	24	m	m	sm	st	sm	st	sm	sm	m	m	m	m	--	1	18S - 1	Ferro et al. (2018)
	*B. cordobae*	Córdoba - Argentina	24	m	sm	sm	sm	sm	sm	sm	m	m	m	m	m	--	--	--	Baraquet et al. (2013)
		Córdoba - Argentina	24	m	m	sm	st	sm	st	sm	m	m	m	m	m	--	11	--	Ferro et al. (2018)
	*B. curupi*	Argentina	24	m	m	sm	st	sm	st	sm	m	sm	sm	m	m	--	1	--	Ananias et al. (2004)
		Misiones - Argentina	24	m	m	sm	st	sm	st	sm	sm	m	m	m	m	--	1	--	Ferro et al. (2018)
	*B. guentheri*	Rio Grande do Sul - Brazil	24	m	m	sm	st	sm	st	sm	m	sm	sm	m	m	--	10	--	Raber et al. (2004)
	*B. joaquini*	Sul do Brazil	24	m	m	sm	st	sm	st	sm	m	sm	sm	m	m	--	1	--	Ananias et al. (2004)
	*B. marginata*	Sul do Brazil	24	m	m	sm	st	sm	st	sm	m	sm	sm	m	m	--	10	--	Ananias et al. (2004)
	*B. marianitae*	Salta - Argentina	24	m	m	sm	st	sm	st	sm	sm	m	m	m	m	--	11	--	Ferro et al. (2018)
	*B. polytaenia*	--	24	m + sm													--	--	Rabello (1970)
		--	24	NI													--	--	Rabello et al. (1971)
		Brazil	24	NI													--	--	Bogart (1973)
		Espirito Santo - Brazil	24	m	sm	sm	sm	sm	st	sm	m	m	m	m	m	--	--	--	Nunes and Fagundes (2008b)
	*B. prasina*	São Paulo - Brazil	24	m	sm	sm	sm	sm	st	sm	m	sm	sm	m	m	--	9	--	Beçak (1968)
		São Paulo - Brazil	24	NI													12/9	--	Baldissera *et al*. (1993)
		Paraná - Brazil	24	m	sm	sm	st	sm	sm	st	m	m	m	m	m	--	12	18S-12/9 5S - 2-5	Present study
	*B. pulchella*	South America	24	m													--	--	Saez and Brum (1960)
		Argentina	24	NI													--	--	Bogart (1973)
		Córdoba - Argentina	24	m	sm	sm	sm	sm	sm	sm	m	m	m	m	m	--	--	--	Baraquet (2013)
		Buenos Aires - Argentina	24	m	m	sm	st	sm	st	sm	m	m	m	m	m	--	12	--	Ferro et al. (2018)
	*B. riojana*	La Rioja - Argentina	24	m	m	sm	st	sm	st	sm	sm	m	m	m	m	--	11	18S - 11	Ferro et al. (2018)
	*B. semiguttata*	Sul do Brazil	24	m	m	sm	st	sm	st	sm	m	sm	sm	m	m	--	1	--	Ananias et al., 2004
	*B. stellae*	Misiones - Argentina	24	m	m	sm	st	sm	st	sm	sm	m	m	m	m	--	1	18S - 1	Ferro et al. (2018)
*B. punctata*	*B. atlantica*	Bahia - Brazil	24	m	sm	st	sm	st	st	st	sm	m	sm	sm	sm	--	10/12	--	Carvalho et al. (2014)
	*B. cinerascens*	Peru	24	NI													--	--	Bogart (1973)
		Amazonas - Brazil	24	m	sm	st	sm	sm	st	sm	m	st	sm	m	sm	--	11	--	Mattos et al. (2014)
		Tungurahua - Ecuador	24	m	m	sm	t	sm	st	sm	m	m	sm	m	m	--	11	18S - 11	Ferro et al. (2018)
	*B. punctata*	Huanuco - Peru	24	NI													--	--	Bogart and Bogart (1971)
		Peru	24	NI													--	--	Bogart (1973)
		--	24	NI													--	--	Anderson (1991)
		Pará - Brazil	24	m	m	sm	sm	sm	st	sm	m	m	sm	m/sm	sm	--	11	18S - 11	Ferro et al. (2018)
*B. semilineata*	*B. boans*	Amazonas - Brazil	24	m	sm	sm	st	st	st	m	st	st	sm	m	m	--	11	--	Mattos et al. (2014)
		Sta Elena de Uairén - Venezuela	24	NI													7	--	Schmid and Steinlein (2016a)
		Sta Elena de Uairén - Venezuela	24	NI													7	--	Schmid and Steinlein (2016b)
		Pará - Brazil	24	m	m	sm	st	sm	sm	sm	sm	m	m	m	m	--	7	18S - 7	Ferro et al. (2018)
	*B. geographica*	Amazonas - Brazil	24	m	m	st	sm	st	st	sm	st	sm	m	m	m	--	1	--	Mattos et al. (2014)
	*B. pombali*	Bahia - Brazil	24	m	sm	sm	m	sm	sm	sm	m	m	m	m	m	--	7	--	Carvalho et al. (2014)
	*B. semilineata*	Espírito Santo - Brazil	24	m	m	st	sm	sm	st	st	sm	st	m	m	m	--	7	18S - 7	Nunes and Fagundes (2008a)
	*B. cf. semilineata*	Pará - Brazil	24	m	m	sm	st	sm	sm	sm	sm	sm	m	m	m	--	7	18S - 7	Ferro et al. (2018)
	*B. wavrini*	Pará - Brazil	24	m	m	sm	st	sm	sm	sm	sm	m	m	m	m	--	11	18S - 11	Ferro et al. (2018)

2N = diploid number; NORs = nucleolus organizer regions; m = metacentric; sm = submetacentric; st = subtelocentric; a = acrocentric; t = telocentric; NI = Not Informed.

*In situ* location of repetitive DNAs is considered an excellent chromosomal marker for genomic comparison (Machado et al., 2020; Azambuja et al., 2022; Deon et al., 2022). Eukaryotic genomes contain a large portion of repetitive DNA sequences (Sumner, 2003). These sequences are presented as repetitive copies that could be arranged in tandem (gene families and satellite DNAs) or dispersed on the chromosomes (transposable elements-TEs) (Sumner, 2003; Meštrović et al., 2015). The 45S and 5S rDNA gene families are commonly used in chromosomal diversification studies (Ferro et al., 2018; Dc *et al.*, 2022).

Tandem satellite-type repeats are categorized based on the size of their repetitive units and are usually grouped into satellite DNA (100-1000 bp), minisatellites (10-100 bp), and microsatellites (SSR - Simple Sequence Repeats - 1-6 bp) (Tautz, 1993; Li et al., 2002). However, this classification is not static since some authors point out that SSRs can integrate satellite sequences when arranged in chromosomes in arrays of thousands to millions of copies (Garrido-Ramos, 2015, 2017). Satellite DNAs are the main component of heterochromatin (John, 1988; Chaves et al., 2004).

*Boana* is assumed to be arranged in seven phylogenetic species groups. Comparative cytogenetic data within and between groups based on *in situ* localization of repetitive DNAs are still lacking, making it difficult to understand the main mechanisms of chromosome evolution. Here, we performed a comparative analysis among *B. albopuctata*, *B. faber*, and *B. prasina*, sampled in the Atlantic Forest from southern Brazil, based on conventional cytogenetic markers and *in situ* localizations using telomere sequence, rDNA gene families, and microsatellite motifs. Thus, the study goals were to infer mechanisms of chromosomal reorganization and dispersion processes of repetitive DNAs among these three species belonging to three different species groups of *Boana*.

## Material and Methods

### Sampled species and cytogenetic preparations

Four male individuals of each of the following species of *Boana* were collected in União da Vitória, Paraná, Brazil (26º13’48” S and 51º05’09” W): *B. albopuctata*, *B. faber*, and *B. prasina*. Voucher specimens were collected under license ICMBio/SISBIO 63336-1, and deposited in the Herpetological collection at Universidade Tecnológica Federal do Paraná, *campus* Francisco Beltrão (RLUTF 1265-1267). This study was authorized by the Ethics Committee of Animal Usage of the Universidade Estadual do Paraná (Process CEUA 2021/0001), and Biosafety Certification according to Comissão Técnica Nacional de Biossegurança - CTNBio (CQB No. 0063/98).

Mitotic chromosomes were obtained from bone marrow using the method of Baldissera Jr. *et al.* (1993), and the slides were stained with 5% Giemsa diluted in phosphate buffer pH 6.8. C-banding was performed using barium hydroxide (5% Ba(OH)_2_ at 25 °C for 3 min), subsequent incubation in salt solution (2×SSC at 60 °C for 30 min), and 5% Giemsa staining (Sumner, 1972). The silver staining consisted of 2 min and 30 s at 60 °C of two parts of a 50% solution of silver nitrate and one part of 2% gelatin/1% formic acid solution (Howell and Black, 1980).

### Obtaining the repetitive sequences and probes

The genomic DNA was extracted from *B. faber* muscle tissue using the Cetyltrimethylammonium bromide (CTAB) method (Murray and Thompson, 1980) and was used as template in Polymerase Chain Reactions (PCR). The 5S rDNA sequence was amplified with the primers 5SA_Fw (5’-TACGCCCGATCTCGTCCGATC-3’) and 5SB_Rv (5’CAGGCTGGTATGGCCGTAAGC-3’) (Martins and Galetti, 1999), and the 18S rDNA sequence was amplified using 18S_Fw (5’ -CCGCTTTGGTGACTCTTGAT-3’) and 18S_Rv (5’-CCGAGGACCTCACTAAACCA-3’) (Gross et al., 2010). In general, the amplification reactions were performed as follows: 40 ng genomic DNA, 0.2 μM forward primer, 0.2 μM reverse primer, 0.16 mM dNTPs, 1U *Taq* DNA Polymerase (Invitrogen, Waltham, MA, USA), and 1.5 mM MgCl_2_ in 1x reaction buffer (200 mM Tris, pH 8.4, 500 mM KCl). The amplification program was as follows: 5 min - 95 °C / 30 cycles (30 s - 95 °C, 45 s - 56 °C, 2 min - 72 °C) / 7 min - 72 °C. PCR products were purified using the GenElute PCR Clean-Up Kit (Sigma Aldrich, St Louis, MO, USA), and cloned using pGEM^®^-T Easy Vector Systems (Promega, Madison, WI, USA). The clones obtained were sequenced using the ABI-PRISM Genetic Analyzer (Applied Biosystems, Carlsbad, CA, USA). The sequences were analyzed in the Nucleotide Basic Local Alignment Search Tool (BLASTn) (Altschul et al., 1990) and Rfam databases (Kalvari et al., 2018).

The general telomeric sequence of vertebrates (TTAGGG)_n_ was generated by PCR in two amplification conditions, using the primers set (TTAGGG)_5_/(CCCTAA)_5_ (Ijdo et al., 1991). The first amplification was performed with low stringency: 4 min - 94 ºC / 12 cycles (1 min - 94 ºC, 45 s - 52 ºC, 1 min 30 s - 72 ºC); followed by 35 cycles of high stringency: 1 min - 94 ºC, 1 min 30 s - 60 °C, 1 min 30 s - 72 °C. The repetitive sequences were labeled in PCR reactions to generate probes. The 5S rDNA was labeled using digoxigenin-11-dUTP (Jena Bioscience, Dortmund, Germany), and 18S rDNA was labeled using biotin-16-dUTP (Jena Bioscience), while for the telomeric sequence, it was used the aminoalyl-dUTP-Cy5 nucleotide (Jena Bioscience). The amplification reactions were performed with the specific primers and the mixtures contained 20 ng DNA, 1 μM of each primer, 40 mM dATP/ dGTP/ dCTP, 28 mM dTTP, 12 mM labeled nucleotide, 1U *Taq* DNA polymerase (Invitrogen), 2 mM MgCl_2_ and 1x reaction buffer. The amplification program: 5 min - 95 °C / 30 cycles (30 s - 95 °C, 45 s - 56 °C, 2 min - 72 °C) / 7 min - 72 °C.

The microsatellites motifs (CA)_15_, (GA)_15_, (CAG)_10_, (CGC)_10_, (GAA)_10_, (GACA)_8_, and (GATA)_8_ were directly labeled with Cy3-fluorochrome (Sigma-Aldrich) at the end 5’ during synthesis.

### *In situ* localization

Fluorescence *in situ* hybridization (FISH) was performed under stringency conditions close to 77% (200 ng of each probe, 50% formamide, 10% dextran sulfate, 2xSSC - saline-sodium citrate; 16 h of hybridization at 37 ºC), according to Pinkel et al. (1986). Fluorescence signals detection was performed using the antibodies streptavidin conjugated with Alexa Fluor 488 (Invitrogen) (18S rDNA recognition) and anti-digoxigenin conjugated with rhodamine (Roche Applied Science, Penzberg, Germany) (5S rDNA recognition). Chromosomes were counterstained with 0.2 μg/mL 4′,6-diamidino-2-phenylindole (DAPI) in Vectashield mounting medium (Vector Laboratories, Burlingame, CA, USA) and analyzed using ZEN digital image capture software coupled to a Carl Zeiss AxioLab A1 microscope. Approximately 30 metaphase cells were analyzed for each probe/specimen. The chromosomal morphology was determined according to the arms relationship criterion proposed by Green and Sessions (1991) (Table S1), and arranged into karyotypes.

## Results

### Karyotype description

**Figure 1-:**
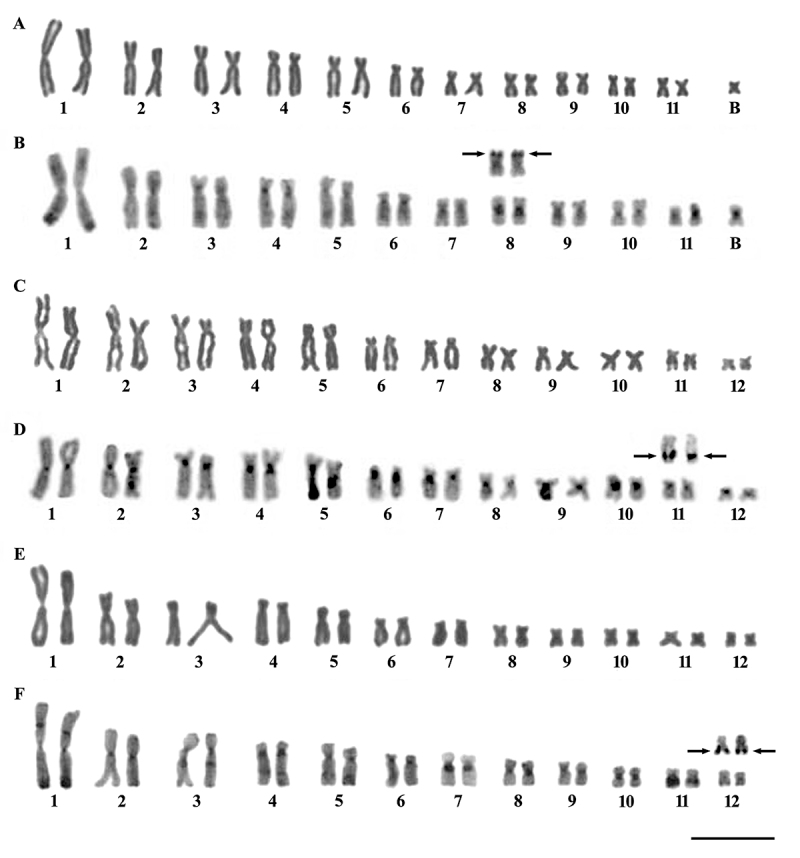
Karyotypes arranged from Giemsa staining and C-banding, respectively: (A, B) *B. albopunctata* with 2n = 22 chromosomes and presence of one B chromosome, (C, D) *B. faber*, and (E, F) *B. prasina*, both with 2n = 24 chromosomes. Above the respective pairs, the NOR-bearing chromosome pairs revealed by silver impregnation (arrows). Bar = 10 µm.

Chromosomal analysis in *B. albopunctata* showed two distinct cytotypes (2n=22 and 22 + 1B), resulting in intra- and inter-individual variations 0-1 B chromosome (Figure 1A and Table 1). *Boana albopunctata* karyotype was arranged in metacentric (m) pairs 1, 2, and 11, submetacentric (sm) pairs 3, 5, 7-10, and subtelocentric (st) pairs 4 and 6, FN=44 (Figure 1A). The extra chromosome (small m B-chromosome) was present in three of the four analyzed specimens, 61.54% on average of the analyzed cells (Table 2). C-banding showed the heterochromatin distributed preferentially on the centromeric regions, besides additional blocks in the terminal regions of the chromosome 1q, interstitial markers in the 1p and in the q arm of chromosome pairs 2 to 7, as well as a conspicuous heterochromatic block in the pericentromeric region of the pair 8 (Figure 1B). Furthermore, constitutive heterochromatin was located on the pericentromeric region of the B chromosome (Figure 1B). *Boana albopunctata* karyotype showed NOR in the terminal region of 8p (Figure 1B).

**Table 2 -:** Frequency of the B chromosome in four *B. albopunctata* analyzed specimens.

Specimen ID	Metaphases N	B frequency
40	22	68.18%
44	21	90.48%
45	8	87.50%
47	18	0%
	**Total = 69**	Average = 61.54%

*Boana faber* showed 2n=24 chromosomes, and the karyotype was arranged in m pairs 1, 2, 8, 10, and 12, sm pairs 3-5, 9, and 11, and subtelocentric (st) pairs 6 and 7, NF=48 (Figure 1C). The heterochromatin was distributed in centromeric bands in all chromosomes of the karyotype, besides interstitial bands on chromosome pairs 2, 3, 5, 6, and 7 (Figure 1D). The NOR site was located on the pair 11q (Figure 1D).

The karyotype of *B. prasina* showed 2n=24 chromosomes, arranged in m pairs 1, 8-12, sm pairs 2, 3, 5, and 6, and st pairs 4 and 7, NF=48 (Figure 1E). The C-banding showed conspicuous terminal chromosome bands on the q arm of pair 1, large pericentromeric blocks of chromosome pairs 4, 7, and 10, and interstitial bands in the p arms of pair 1 and q arm of the chromosome pairs 3 to 5 (Figure 1F). Additionally, pair 11q presented a conspicuous interstitial heterochromatic block (Figure 1F). *Boana prasina* karyotype showed NOR on the terminal region of the 12q (Figure 1F).

### Chromosomal mapping of repetitive sequences

**Figure 2 -:**
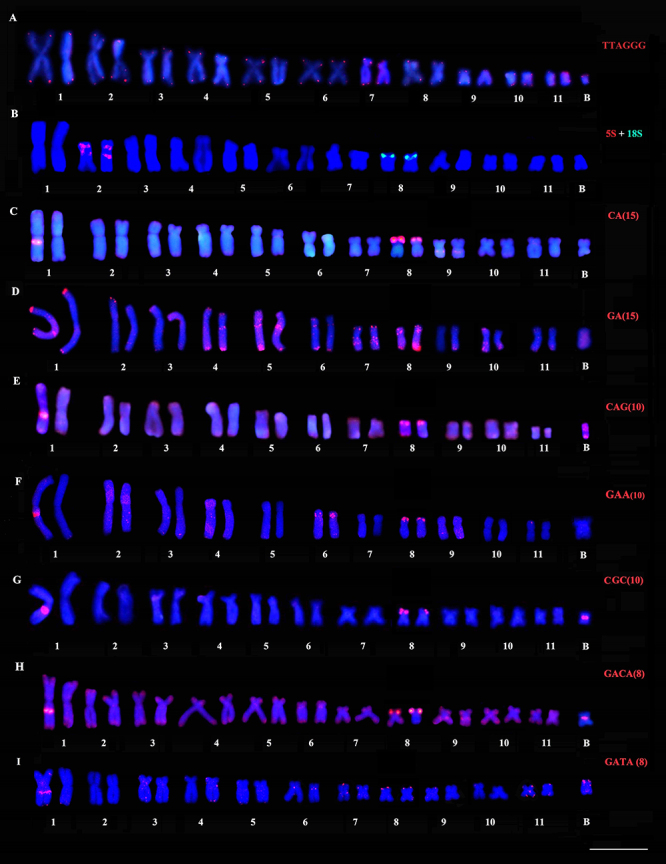
Karyotype of *B. albopunctata* submitted to FISH with the following repetitive sequences: (A) telomeric probe, (B) ribosomal probes, and (C-I) microsatellite sequences. Bar = 10 µm.

In *B. albopunctata*, the *in situ* location of the telomeric sequence was restricted to the terminal regions of all chromosomes (Figure 2A). Double FISH using rDNA probes showed interstitial 5S rDNA sites on both arms of chromosome 2, and the 18S rDNA cluster in the terminal region of the 8p (Figure 2B). The microsatellite repeats (CA)_n_, (GA)_n_, (CAG)_n_, (CGC)_n_, (GAA)_n_, (GACA)_n_, and (GATA)_n_ showed hybridization signals on the *B. albopunctata* karyotype (Figure 2C-I, respectively). Conspicuous markings of all microsatellites were detected in the interstitial position of one homologous of pair 1 and the terminal region of the 8p (Figure 2C-I). In addition, (CA)_n_ motifs were evidenced in interstitial region of 9q (Figure 2C). The (GA)_n_ signals were detected in the terminal region of most chromosomes, at the proximal region of the q arm in pairs 4 and 5, in the interstitial region of the q arms of pairs 7 and 8, and the terminal region of 8q (Figure 2D). The microsatellite (CAG)_n_ was located in the terminal regions of the chromosomes, including the B chromosome, which also presented accumulation in its pericentromeric region (Figure 2E). (GAA)_n_ motifs were detected in the interstitial region of the pair 6p, in addition to dispersed signals along the chromosomes 2, 3, 4, and 9 (Figure 2F). The location of the (CGC)_n_ repeat also coincided with the heterochromatin in the pericentromeric region of B chromosome (Figure 2G). The (GACA)_n_ tetranucleotide was mapped in the terminal regions of all chromosome pairs, the interstitial region of the pair 9q, and the pericentromeric region of B chromosome (Figure 2H). The (GATA)_n_ sequence showed hybridization signals in the terminal region of the p arm of the B chromosome and dispersed markings in pairs 3, 4, 6, 7, and 11 (Figure 2I).

**Figure 3 -:**
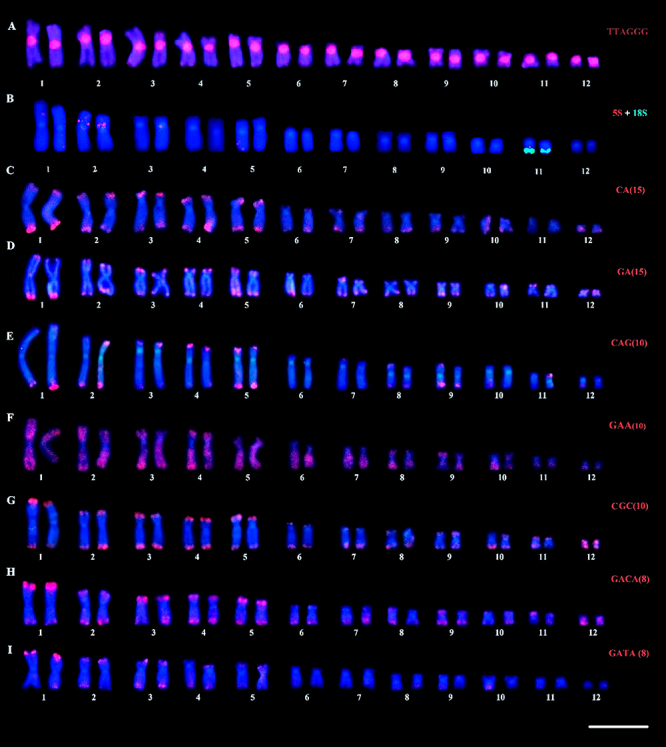
Karyotype of *B. faber* submitted to FISH with the following repetitive sequences: (A) telomeric probe, (B) ribosomal probes, and (C-I) microsatellite sequences. Bar = 10 µm.

In *B. faber*, the (TTAGGG)_n_ probe was located in the telomeric region, in addition to accumulations in the pericentromeric region of all chromosomes (Figure 3A). Double FISH with the rDNA probes detected the 5S rDNA cluster in an interstitial position in pair 2p, while the 18S rDNA was located in the terminal region of the q arm of pair 11 (Figure 3B). *In situ* localization of the (CA)_n_, (GA)_n_, (CAG)_n_, (CGC)_n_, (GAA)_n_ and (GACA)_n_ microsatellites revealed signals preferentially located at the terminal regions, besides signals scattered along the chromosomes (Figure 3C-H, respectively). Except for the centromeric and proximal regions, the microsatellite (GAA)_n_ showed a dispersed pattern distribution along the chromosome arms (Figure 3F). (GATA)_n_ motifs were *in situ* located preferentially on the terminal regions of chromosome pairs 1, 2, 3, 4, 5, and 10 (Figure 3I).

**Figure 4 -:**
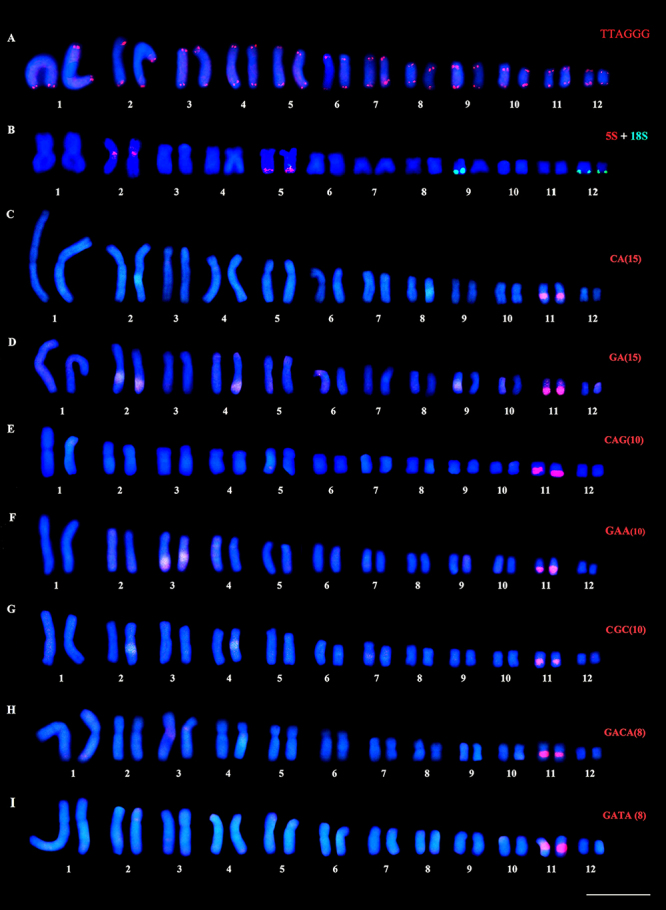
Karyotype of *B. prasina* submitted to FISH with the following repetitive sequences: (A) telomeric probe, (B) ribosomal probes, and (C-I) microsatellite sequences. Bar = 10 µm.

The (TTAGGG)_n_ sequence was detected in the terminal regions of all chromosomes of *B. prasina* (Figure 4A). Double FISH detected the 5S rDNA cluster on the centromeric region of pair 2 and in the terminal region of the 5q, while the 18S rDNA probe hybridized in the terminal region of the q arm of pair 12 and only one homologous of pair 9 (Figure 4B). All the microsatellite repeats analyzed (CA, GA, CAG, CGC, GAA, GACA, and GATA) hybridized exclusively to the q arm of pair 11 (Figure 4C-I, respectively). 

### Analysis of rDNA sequences

The *B. faber* 5S rDNA sequence comprises 219 bp, 85.22% identity with 5S rRNA from *Rana temporaria* (XR_005742848.1), and E-value of 2e-24 with the 5S ribosomal RNA in Rfam. The non-transcribed region (NTS) corresponds from nucleotide 1 to 97 and the transcribed region from 98 to 219. The partial sequence of *B. faber* 18S rDNA comprises 989 bp, 95.49% identity with *Boana boans* 18S rDNA (EF376085.1), and E-value of 9.5e-224 with eukaryotic small subunit ribosomal RNA in Rfam. The sequences were deposited in GenBank (IDs: ON809568 and ON809569, respectively).

## Discussion

### Numerical chromosome changes in *Boana*

The *Boana* genus is organized into seven phylogenetic species groups (Faivovich et al., 2005). Except for the members of the *B. benitezi* group, for which cytogenetic data are not available so far, the species already karyotyped from the *B. faber*, *B. pellucens*, *B. pulchella*, *B. punctata*, and *B. semilineata* groups, presented 2n=24 chromosomes, including *B. faber* and *B. prasina* described in this study (Table 1). Despite 2n conservation among these species, morphological chromosome alterations changing the karyotypic formulas occurred independently in each species group lineage (Table 1). 

On the other hand, *B. albopunctata*, *Boana cf. alfaroi*, *B. leucocheila*, and *B. multifasciata* have 2n=22 (Ferro et al., 2018, and references therein). End-to-end chromosome fusion, or reciprocal translocation involving the smallest pairs (NOR-bearing), has been proposed to explain the numerical chromosomal reduction observed in *B. albopunctata* species group, considering 2n=24 as a putative plesiomorphic condition in *Boana* (Bogart, 1973; Gruber et al., 2007). Based on this assumption, the NOR site is repositioned from chromosome 11 to 8 in species with 2n=24 and 2n=22, respectively.

According to previous assumptions, the origin of a small B metacentric in *B. albopunctata* appears as a subproduct of this numeric chromosomal reorganization (Bogart, 1973; Gruber et al., 2007). Although the NOR location on pair 8 is conserved in species with 2n=22 and on pair 11 or 12 in species with 2n=24 in the group *B. albopunctata*, the NORs showed chromosomal repositioning in other groups of *Boana*, without changing the 2n (see Table 1). Also, pairs 11 and 12 in karyotypes with 2n=24 of the *B. albopunctata* species group are usually m or sm chromosomes, indicating a more complex mechanism for chromosome number reduction. Thus, although the fusion between pairs 11 and 12 proposed by Gruber *et al.* (2007) may be parsimonious in explaining the origin of 2n=22, the breakpoints and mechanisms related are not fully understood. Besides that, no ITS vestiges were observed in the analyzed *B. albopunctata* karyotype, suggesting the occurrence of double-strand breaks in the origin of chromosomal fusion.

Only some populations of *B. leucocheila* and *B. albopunctata* carry B chromosomes (Table 1), similar in size and metacentric morphology (Gruber et al., 2007; Ferro et al., 2018). In *B. albopunctata*, when the B chromosomes are present, in all cases are metacentric small-sized but with distinct levels of heterochromatinization (Gruber *et al.*, 2007; Ferro *et al.*, 2012). These findings, as observed in *B. albopunctata* analyzed, indicate a population differentiation of the B chromosome by progressive DNA repeats accumulation.

Using a chromosome probe obtained from the microdissection of a B chromosome of *B. albopunctata*, Gruber et al. (2014) observed hybridization signals just on the supernumerary. Based on the B chromosome painting data, Gruber *et al.* (2014) suggested a composition enriched with repetitive DNA and an interspecific origin of the B. In the present study, FISH experiments with microsatellite probes showed that the pericentromeric region of the B chromosome is enriched with CGC and GACA repeats, and in the terminal regions, there are CAG and GATA accumulations. These microsatellites are also accumulated in pair 8. Based on this evidence, we suggest that the B chromosome could have originated from an A set chromosome, microsatellite enriched, such as the pair 8. However, future genomic studies allied to chromosome painting and repetitive DNA probes from B are required to elucidate the mechanism of origin of the B chromosome in these species.

### Chromosome mapping

In Hylinae, NORs located on a small-sized chromosome are common in their representatives, suggesting a homeology involving the NOR-bearing chromosomes (Cardozo et al., 2011; Catroli et al., 2011). Most species of *Boana* share the putative NOR plesiomorphic condition (on pair 11), although in some species of the *B. albopunctata*, *B. pulchella*, and *B. semilineata* groups, the locus occurs in a higher size chromosome (Table 1). Multiple NORs, *i.e.*, on two chromosome pairs, were detected only in *B. atlantica* and *B. prasina* karyotypes (Baldissera *et al.*, 1993; Carvalho et al., 2014). The chromosomal dynamics of NOR location in anurans may be the result of intra and inter-chromosomal rearrangements, like inversions, fusions, and translocations, by TE-mediated transpositions events or reinsertion of errors during amplification events (Schmid et al., 1995; Kaiser et al., 1996; Lourenço et al., 2000; Huang et al., 2008; Cazaux et al., 2011; Ferro et al., 2018; Deon et al., 2022). In the three *Boana* species analyzed, the NORs were located in usual chromosome positions for each species, previous corroborating studies (Gruber et al., 2007; Carvalho *et al.*, 2014; Schmid and Steinlein, 2016a). *Boana prasina* presented an additional 45S rDNA site on the karyotype, as also observed by Baldissera *et al.* (1993), but a non-active nucleolus. A detailed explanation of silent NOR was described in *Arabidopsis* genome, where NOR silencing appears to be controlled by sequences outside the rDNA array (McStay, 2016). This finding indicates that a rDNA unit transposition not carrying their transcription regulators could imply non-activation.

Here we report, for the first time, the physical mapping of 5S rDNA loci in species of *Boana*. In other anurans, the location of the 5S rDNA tends to be conserved in the karyotypes of the species (Vitelli et al., 1982; Rodrigues et al., 2012). The three *Boana* species analyzed shared the chromosome location of 5S rDNA cluster. Furthermore, *B. albopunctata* and *B. prasina* showed additional 5S rDNA sites. The 5S rDNA clusters were considered unstable genomic regions in some groups, subjected to double-strand breaks and chromosomal rearrangements, promoting karyotypic remodeling (Glugoski et al., 2018; Deon et al., 2020, 2022). These additional sites in *Boana* suggest that the 5S rDNA family was also subjected to transposition or translocation events of repetitive sequences in these karyotypes.

The distribution of heterochromatic bands tends to be quite diverse among the karyotypes into the distinct species groups of *Boana* (Baldissera *et al.*, 1993; Gruber et al., 2007; Carvalho et al., 2009, 2014; Ferro et al., 2018). Heterochromatin features, such as position, amount, and DNA repeat units, were efficient chromosome markers to evaluate the karyotype diversification in the *Boana* studied species. The extensive heterochromatic blocks presented in some chromosome pairs indicate repeat unit amplification, reinforcing the role of the repetitive DNAs in chromosome evolution in *Boana*.

The telomeric sequence distribution on *B. faber* karyotype illustrates the repetitive DNAs potential in minor changes in *Boana* karyotypes. Given the maintenance of 2n=24, chromosomal fusions cannot explain the origin of the ITS observed in the *B. faber* karyotypes (Schmid and Steinlein, 2016b). In some vertebrates, telomeric-like sequences may be found in satellite DNA (Meyne et al., 1990; Garrido-Ramos et al., 1998; Schmid *et al.*, 2014; Schmid and Steinlein, 2016b). Moreover, according to Schmid and Steinlein (2016b), the high intensity of (TTAGGG)_n_ sequences in the heterochromatic pericentromeric area of *B. faber* shows that these repeats are part of centromeric satellite DNA. So, the intense accumulation of pericentromeric (TTAGGG)_n_ sequences in *B. faber* karyotype is an apomorphic feature due to repetitive DNA units’ diversification. 

Ferro et al. (2018), characterizing AT/CG-rich regions, demonstrated the dynamic of heterochromatic domains in *Boana*, and reinforced the need for repeat unit localization to compare heterochromatic blocks in chromosome diversification. In this study, the comparative *in situ* localization of seven microsatellites in *B. albopunctata*, *B. faber*, and *B. prasina* karyotypes revealed genomic differences in the composition of heterochromatin blocks. Despite these species belong to different taxonomic groups of *Boana*, this finding reinforces a significant diversification in their repetitive DNA content.

Some studies have reported that microsatellite sequences are not randomly distributed in eukaryotic genomes, and closely related species tend to have the same chromosomal locations (Cuadrado and Jouve, 2007; Ruiz-Ruano et al., 2015; Zheng et al., 2016; Utsunomia et al., 2018). On the other hand, different patterns in the location of microsatellite repeats may indicate karyotypic diversification in specific lineages, which is occasionally linked to chromosomal rearrangements (Farré et al., 2012; Glugoski et al., 2022). As the species studied here belong to different *Boana* groups (Faivovich et al., 2005), the distribution of microsatellites in the karyotypes confirms distinct chromosomal organizations.

Significant microsatellite sequence accumulations in euchromatic regions, such as those found in *B. albopunctata*, are uncommon. In this species, the seven microsatellites revealed specific sites in the euchromatic segment in only one homologous member of pair 1. Specific accumulations of microsatellites are usual in heteromorphic sex chromosomes due to the emergence of the non-recombinant region (Schemberger et al., 2019). Thus, the association of this heteromorphic region as polymorphic or associated with sex should be further investigated in *B. albopunctata*. However, this pattern of microsatellite organization in the euchromatin was also observed in the karyotypes of other vertebrates, non-related to the sex, as in Cheloniidae (Machado et al., 2020) and Cycloramphidae species (Bueno et al., 2021). Still, the absence of available genomic information does not allow us to understand the structure and functions of these regions. In addition, the colocalization of microsatellites with the NOR can be explained by the presence of repetitive DNAs in the intergenic spacer (IGS) regions (Ruiz-Ruano et al., 2015; Ernetti et al., 2019).

In the *B. faber* karyotype, the GAA motif showed a dispersed and interspaced pattern. The distribution of microsatellite sequences throughout genomes has been associated with the activity of TEs, which may contain microsatellite repeats in its sequences, thus contributing to units spread during transposition events (Akagi et al., 2001; Coates et al., 2010; Pucci et al., 2016). In this way, the GAA expansion could be disseminated into *B. faber* genome as part of a TE. On the other hand, all microsatellite motifs mapped in *B. prasina* showed hybridization signals exclusive and coincident with a heterochromatic block in the long arm of the pair 11. According to Ferro et al. (2018), this heterochromatic block probably represents a synapomorphy within the *B. pulchella* group, which currently includes *B. prasina* and 37 other species (Faivovich et al., 2021). These data suggest extensive actuation of repetitive DNAs in minor chromosomal changes promoting independent diversification in the distinct phylogenetic groups of *Boana*.

## Conclusion

The obtained comparative chromosome analysis revealed that the karyotypes of *B. albopunctata*, *B. faber*, and *B. prasina* presented intrinsic differences, mainly related to the presence of the B chromosome, the location and number of rDNA sites, and the dispersion pattern, and location of microsatellite units. These findings revealed karyological diversification among the species belonging to *Boana* taxonomic groups, which may be associated with the dispersion of repetitive DNAs, promoting changes in morphology and composition of the chromosomes.

## Supplementary material

The following online material is available for this article:

Table S1 - Chromosome measurements of *Boana* species of the present study.
