# Supplementary material for: Comparative cytogenetics among Boana species (Anura, Hylidae): focus on evolutionary variability of repetitive DNA
Source: Genet Mol Biol. 2023 Jan 6;45(4):e20220203. doi: 10.1590/1678-4685-GMB-2022-0203 (PMC9827724; doi:10.1590/1678-4685-GMB-2022-0203)
Supplement: Table S1 - [file 1415-4757-GMB-45-4-e20220203-s1.pdf]

Genetics and Molecular Biology
DOI: https://doi.org/10.1590/1678-4685-GMB-2022-0203

Supplementary Material to "Comparative cytogenetics among *Boana* species (Anura, Hylidae): focus on evolutionary variability of repetitive DNA"

Table S1. Chromosome measurements of *Boana* species of the present study. Chromosome percentage relative to the haploid set. Centromeric index ± standard deviation. m: metacentric; sm: submetacentric; st: subtelocentric.

**Species**	**Chromosome pair**												
	**1**	**2**	**3**	**4**	**5**	**6**	**7**	**8**	**9**	**10**	**11**	**12**	**B**
B. albopunctata	18.67 **(m)**<br>0.49 ± 0.04	13.02 **(m)**<br>0.40 ± 0.02	11.79 **(sm)**<br>0.31 ± 0.01	10.81 **(st)**<br>0.23 ± 0.01	9.58 **(sm)**<br>0.26 ± 0.02	7.86 **(st)**<br>0.22 ± 0.01	5.65 **(sm)**<br>0.35 ± 0.03	5.41 **(sm)**<br>0.36 ± 0.01	5.41 **(sm)**<br>0.36 ± 0.01	4.67 **(sm)**<br>0.32 ± 0.02	4.42 **(m)**<br>0.39 ± 0.01		2.70 **(m)**<br>0.45 ± 0.00
B. faber	15.67 **(m)**<br>0.48 ± 0.03	13.91 **(m)**<br>0.40 ± 0.01	11.92 **(sm)**<br>0.26 ± 0.01	10.82 **(sm)**<br>0.29 ± 0.02	10.15 **(sm)**<br>0.33 ± 0.01	7.73 **(st)**<br>0.23 ± 0.02	6.84 **(st)**<br>0.23 ± 0.00	5.52 **(m)**<br>0.44 ± 0.02	5.08 **(sm)**<br>0.35 ± 0.02	5.08 **(m)**<br>0.48 ± 0.01	4.64 **(sm)**<br>0.29 ± 0.02	2.65 **(m)**<br>0.42 ± 0.02	
B. prasina	20.68 **(m)**<br>0.47 ± 0.01	13.09 **(sm)**<br>0.36 ± 0.04	11.26 **(sm)**<br>0.33 ± 0.03	11.26 **(st)**<br>0.19 ± 0.01	9.42 **(sm)**<br>0.31 ± 0.01	6.81 **(sm)**<br>0.31 ± 0.04	6.54 **(st)**<br>0.28 ± 0.01	5.24 **(m)**<br>0.45 ± 0.03	4.45 **(m)**<br>0.47 ± 0.02	4.19 **(m)**<br>0.44 ± 0.02	3.93 **(m)**<br>0.47 ± 0.02	3.14 **(m)**<br>0.50 ± 0.01	
